# Improving contraceptive care for minors in Israel: practice, policy, and training gaps among OBGYNs

**DOI:** 10.1186/s13584-024-00638-4

**Published:** 2024-09-26

**Authors:** Maya Peled-Raz, Orly Goldstick

**Affiliations:** 1 School of Public Health, Faculty of Social Welfare and Health Sciences, University of Haifa, Haifa, Israel; 2 Clalit Health Services, Haifa and Western Galilee District, Haifa, Israel

**Keywords:** Contraceptives, Legal capacity, Parental consent, Mature minor, Ethics

## Abstract

**Background:**

Sexually active adolescents sometimes seek contraceptives without parental consent, posing challenges due to minors' confidentiality and consent regulations. This is especially the case under the un-nuanced Israeli legal scheme regarding adolescents' care.

**Methods:**

Israeli OBGYNs were contacted through mailing lists and social media groups and asked to fill an online questionnaire regarding their experience and protocols concerning prescription of contraceptives to minors. They were also asked about their comprehension of the relevant legal obligations, the importance they ascribe to different ethical interests and considerations, as well as their training.

**Results:**

Of the 177 responding gynecologists, 132 (74.58%) consulted minors about contraceptives during the past year, regardless of a vast lack of training on providing care to minors. More than a third of respondents believed that there is no legal requirement to involve parents in the process, and only 8% assumed a legal obligation for parental involvement in all minors under the age of 18. Three quarters would "almost always" prescribe contraceptives without parental knowledge, if requested, while 20% never would. No correlation was found between respondents' practices and their perception of the relevant legal obligations. Participants agreed that the risk to the health of the minor as a result of having sex without contraceptives is of utmost importance. Yet, those willing to prescribe gave greater weight to this consideration, while those who do not prescribe were more concerned with the legal ramifications of such an act. The majority identified the age of 15 as the threshold for consistently prescribing contraceptives to minors without parental involvement.

**Conclusion:**

This study highlights the significant gaps in both the legal framework and the training of Israeli OBGYNs, and further supports confidential prescription of contraceptives to minors 15 years and older, via Article 6 of the Israeli Legal Competence and Guardianship Law. Legislative reform, professional guidelines and education and training programs are all needed to ensure consistent and legally sound practices, that safeguard the health and rights of minors. It is imperative to guide healthcare providers, including OBGYNs prescribing contraceptives to minors, on managing the care of minors refusing parental involvement, clarifying the legal framework and ethical considerations involved.

**Supplementary Information:**

The online version contains supplementary material available at 10.1186/s13584-024-00638-4.

## Introduction

### Adolescents' sexual reproductive healthcare needs and barriers

Sexual activity in adolescence is a common phenomenon in Europe, ranging between a reported 30% of all 15-year-old youths in Bulgaria, to around 7% of all 15-year-old youths in Kazakhstan [6].

Based on the latest WHO-Israel Health Behaviors in School-Aged Children survey [6], conducted with 10th and 12th graders attending secular schools, 20% of Israeli high school students had full sex during their lifetime, with around 10% of all girls. Adolescence sex is more prevalent amongst Jewish girls (14%; 4% for Arab girls) and rises with age: around 6% of 10th grade Jewish girls attested to ever having full sex, rising to 18.5% in the 12th grade.

About 75% of the students who reported having sex during their lifetime, reported that they used a condom the last time they had sex, and about 36% reported that they had used a birth control pill the last time they had sex (with similar results for Jews and Arabs).

While the necessity to attend to the sexual-reproductive medical needs of adolescents is thus clear, adolescence sexual health care in Israel, as in other countries, encounters severe barriers. These include adolescents' inexperience and lack of knowledge about accessing health care, and—most relevant in the face of sexual reproductive topics—heightened sensitivity to confidentiality breaches and capacity-restrictive legislative frameworks [10]. Adolescents often cite concerns about confidentiality as a significant obstacle to accessing healthcare services [13]. They are more inclined to seek medical assistance from healthcare providers who guarantee privacy, sometimes even opting to forgo healthcare altogether, to avoid their parents discovering their medical concerns [4].

Essentially, when an adolescent girl visits a gynecologist for contraceptive care, she demonstrates maturity and responsibility, surpassing the common lack of experience and knowledge. On occasion, the girl may attend the clinic without her parents' knowledge, choosing not to disclose her health need to them. In such cases, confidentiality and parental consent regulations may stand in the way of the physician's ability to administer care, endangering both the girl's health and her trust in the health system.

## The "mature minor" principle

Studies show that a significant number of individuals below the age of 18 exhibit a level of decision-making capability comparable to that of adults. However, the age at which a minor achieves this competence varies from individual to individual [7]. When evaluating a minor's capacity to make decisions regarding their health, two factors should be considered: an objective assessment of the minor's age and a subjective evaluation of their comprehension and maturity level. This subjective evaluation is based on criteria derived from psychological theories and studies [14].

In accordance with American law, the rights of minors are categorized and assessed based on distinct subgroups, taking into consideration the minor's abilities relative to their skills and life circumstances. 'Small Minors' refers to children of a young age who rely heavily on their parents. It is presumed that their level of comprehension is basic and may be inadequate to fully grasp explanations regarding a given medical condition [8]. 'Mature minors' encompass teenage individuals who demonstrate emotional and mental maturity, enabling them to comprehend the offered treatment and associated risks. This then allows them to make informed and prudent decisions regarding their healthcare [29]. 'Emancipated minors' typically refer to those who do not reside with their parents and lead an independent lifestyle [28]. Emancipated minors possess the exclusive authority to consent to their own medical treatment.

Over the years, the age at which minors were considered mature enough to consent to medical treatment on their own (as opposed to giving non-legally binding Assent) has declined, especially when relating to decisions regarding termination of pregnancy and contraceptive use [1, 11]. Over the past 30 years, 21 of the American states have allowed all minors to receive contraception without parental consent or knowledge, and 14 states have established similar rights for married or adult minors. All 50 states and the District of Columbia allow minors to be tested for sexually transmitted diseases and receive relevant treatment without parental involvement [5].

English law recognizes the legal capacity of a 16-year-old minor to consent to any medical treatment [31], Section 8]. It then distinguishes between minors with decision-making ability and minors who lacks such ability, to be determined by the minor's physician and influenced by the age of the minor and the nature of the medical treatment required [9]. English law follows the Gillick precedent, according to which parental authority decreases in direct proportion to the increase in the age and maturity of the child [32]. The Gillick ruling was a breakthrough for the recognition of children's rights in England and paved the transition from a discourse of "the best interests of the child" and parental authority to a discourse of "children's rights".

In Spain and Scotland, minors aged 16 and above are eligible to provide consent. In Germany, maturity is assessed on a case-by-case basis. In Finland and Sweden, minors can consent based on their maturity, even though legal adulthood is typically at 18 years. In Denmark, minors above 15 years old may consent if they demonstrate maturity; in Norway, the age is over 16 years, except under specific circumstances, and in Iceland, it's at 16 years old. In Italy and France, individuals attain the capacity to act in health-related matters upon reaching the legal age of majority, which is 18 years. Regardless of age, minors are entitled to information, participation, and the opportunity to express (non-legally binding) assent, with recourse to a guardian or judge in case of disagreement [2].

## Minors' (in)ability to consent to treatment under the Israeli legal scheme

On August 4, 1991, the State of Israel ratified the UN Convention on the Rights of the Child, committing to guarantee children, able to express their own opinion, the right to express such opinion freely, as well as give due weight to it, in accordance with their age and maturity [30]. In light of this, it is reasonably expected that the state and its authorities will encourage a policy that supports the provision of information to minors, as well as their participation in decision-making, in a manner consistent with their developing capabilities [8]. Regardless, the State of Israel has so far refrained from coherently regulating the status of the mature minor in health-related decision-making processes.

According to Article 13(a) of the Israeli Patient's Rights Law [22], patients will not be given medical treatment without their informed consent. For the patient's consent to be considered "informed", it must meet three threshold conditions: the patient must be competent, and his consent must be free from undue pressure, as well as based on such information as reasonably required by the patient, in order to make the decision. According to the Israeli Legal Capacity and Guardianship Law [21], a girl under the age of 18 is *a minor* (Article 3), her parents are her natural guardians (Article 14), and any "legal act" on her part requires their approval (Article 4). Consent to medical treatment is a "legal act" and therefore a minor is not legally competent to independently decide whether to receive (or refuse) medical treatment, including contraceptive measures. While minors may "assent" to treatment (i.e. express their will to be treated) alongside their parents' "informed consent", they may not themselves give "informed consent" (nor "informed refusal")—in its legally binding and authorizing sense. This legal stance does not differentiate between different "types" of minors. The notion of a "mature minor" is absent from the Israeli legal framework altogether, and thus minors of all ages and maturity must adhere to the same parameters of the law.

Israeli law does bestow explicit legal powers upon minors for a select set of treatments, either alongside or regardless of their parents' stance on the matter. Those treatments are presented in Table 1.

**Table 1:** Exceptions to the Israeli "minors' legal incapacity" rule in the medical context

Treatment	Capacity	Age
Termination of pregnancy (Penal [25]. Articles 312–321)	Capacity to consent	Any
HIV testing (Detection of HIV Virus in Minors Law, 1996) [16]	Capacity to consent	14 +
Psychiatric hospitalization (Youth Law (Care and Supervision) 1960) [27]	Minor's consent required alongside parental consent. Without it—a court order will be required	15 +
Genetic testing (Genetic Information Law, 2000) [18]	Minor's consent required alongside parental consent. Without it—a court order will be required	16 +
Treatment of the "dying patient"(The Dying Patient Act, 2005) [17]	To choose to be treated—regardless of parental position	Any
	To refuse to be treated, supported by a decision by an Ethics committee, regardless of parental position	15 +
	Capacity to Consent	17 +

Minors' general legal incapacity to make medical decisions stands in stark contrast to their legal standing in other Israeli contexts, such as Article 13 of the Penal Code [Penal [25]] and Article 9 of the Torts Ordinance [26], which establish that minors bear criminal responsibility and tort liability for their actions from the age of 12. Moreover, minors can provide medical assistance as volunteer medics before attaining the autonomy to make decisions regarding their own health [12]. Compounding this issue is the lack of acknowledgement for "emancipated minors" in Israel. Consequently, teenage mothers take on guardianship duties for their children while still under their parents' guardianship.

### Section 6 of the legal capacity law and its application to contraceptive care

Article 6 of the Israeli Legal competence and Guardianship Law [21] states that a minor's legal action, which is of a type commonly performed by minors of his age, cannot be annulled, albeit the absence of parental consent, lest it caused real damage to the minor or his property. This allows for some recognition of an interpretive legal capacity to minors, which is dependent on cultural and generational contexts [7].

Over the years, many Israeli minors have begun to seek medical treatment in its various forms, unaccompanied by their parents [9]. In 1997, the Ministry of Health's legal counselor explicitly addressed, albeit informally, the issue of caring for minors who refuse parental involvement. It interpreted Article 6, as allowing, and even expecting caregivers to determine "for example in the case of girls seeking contraceptive counseling, whether 16-year-old girls would commonly seek such counseling on their own". If the answer is yes, and one believes that the girl is mature enough to understand the risks and benefits of treatment, the caregiver may give her treatment without parental involvement and consent [15].

In 2004, the Head of the Ministry of Health's (here after—MoH's) Medical Administration guidelines titled "Visits of Unaccompanied Minors to Primary Care Clinics" [19]. Explicitly based on Article 6, this circular grants caregivers within a primary care clinic the authority to administer "routine" medical treatment to a minor aged 14 or older (and in exceptional cases, even younger) without necessitating explicit parental consent or the presence of one's parents. This authorization relies on the minor's personal informed consent to be examined or treated. The caregiver must be assured that the minor possesses the cognitive and emotional capacity to comprehend the necessary information for making an informed decision about the treatment [12].

It is important to underscore that this protocol pertains exclusively to visits to primary care clinics, involving minors who are familiar (either personally or through their family) to the clinic's medical personnel, and exclusively for routine treatments and examinations.

Also, the circular requires the caregiver to inform the minor's parents of the results of the examination and treatment retroactively, except in cases where the minor "strongly objects" to his parents' knowledge of the treatment, or the caregiver himself believes that parental involvement may be detrimental to the minor or his compliance with treatment. In such a case, concealment of the information should be accompanied by the involvement of a youth welfare worker (Section 8.4 of the circular). Interestingly, this circular marks the firsts instance, in which the MoH explicitly recognizes situations where it may be appropriate to withhold information from a child's parents regarding their treatment. Nevertheless, the prevailing interpretation of the circular does not view a visit by a minor to a gynecologist as a visit to a "primary care clinic", and it is unclear whether use of contraceptives should be viewed as a "routine treatment". Hence, counseling and giving contraceptive pills to a minor, without obtaining parental consent, should probably not be viewed as comprehensively authorized by virtue of this circular.

In 2010, the Ethics Bureau of the Israeli Medical Association (IMA) released a position paper titled "The Rights of Minors in Medical Treatment." This paper delved into various aspects of minors' medical treatment, including situations where a minor declines parental involvement in their care [20]. According to the Ethics Bureau, if a doctor attempts to persuade the minor to involve a parent but the minor still "firmly declines", there are ethical considerations that may warrant the doctor treating the child while maintaining confidentiality. This decision would be made at the doctor's discretion, considering the specific circumstances of the case and weighing the potential harm that could arise from a breach of confidentiality.

It is important to note, that while the recognition of minors' legal authority to give informed consent, under the 2004 circular, is granted based on considerations relating to his or her maturity and abilities, both the circular and the IMA position paper base the authority to occasionally conceal the treatment from one's parent on the best interests of the child—and not on one's evolving capacities and rights.

## Methods

*Aim*: To assess practices and knowledge of Israeli Obstetricians and Gynecologists regarding prescription of contraceptives to minors without parental knowledge nor consent.

*Sample and research tools*: This study employed a non-probability snowball sampling approach. All OBGYNs practicing in Israel were eligible for inclusion. No exclusion criteria were employed. Participants were contacted through the mailing list of the Israeli Society for Community OBGYN (n = 250 members) as well as other professional email, WhatsApp and social media groups, providing a link to a Google Forms questionnaire. They were encouraged to share the survey with their colleagues. Participation was entirely voluntary, and measures were taken to safeguard respondent anonymity, to further the authenticity of their input.

Following the Checklist for Reporting Results of Internet E-Surveys (CHERRIES) guidelines [3], the questionnaire was preceded by an informational introduction, outlining the target population, study rationale, and content. Participants were assured of anonymity, informed that the survey would take approximately 10 min to complete, and that participation was entirely voluntary. To ensure informed consent, respondents were instructed to proceed with the survey only if they agreed to participate.

The questionnaire utilized fixed questions, incorporating adaptive questioning processes. It was administered in Hebrew. Given the noninvasive nature of the questionnaire, non-response options were not provided. No cookies were utilized, and IP addresses of client computers were not collected. Upon completion of the survey, the data were downloaded and permanently deleted from Google Forms.

As no validated tools were found pertaining to this subject, our questionnaire was developed based on clinical expertise. Content and clarity validation was achieved via a focus group of 3 practicing OBGYNs. The questionnaire was designed to probe various facets of prescribing contraceptive pills to minors without parental involvement and consisted of 4 parts. The first (9 questions in total), encompassed inquiries about the respondent’s empirical experience, including the volume of minor patients they attended to in the preceding year, and the prevalence of minors requesting confidential contraceptive prescriptions. Additionally, the survey inquired into the respondent's existing protocols concerning documentation and communication with contraceptives seeking minors. It sought to uncover whom physicians typically consulted in these scenarios and gauged their comprehension of the legal obligations associated with such prescriptions. This included exploring their knowledge of the extent of parental access to minors’ medical records via Health Maintenance Organizations' (HMO) online apps.

The second section, consisting of two questions, aimed to chart the participants' perception of the legal obligations pertaining to the care of minors in the absence of parental involvement. Specifically, it sought to understand their interpretations of what the law and guidelines dictated in such situations.

In the third part (3 multi-layered questions) participants were asked to articulate their viewpoints on a spectrum of ethical and professional concerns linked to the confidential prescription of contraceptives to minors, including the desired nature of parental involvement, when and under what conditions it was justified to forgo parental involvement, as well as the importance they attribute to different relevant ethical interests and considerations.

We also compiled comprehensive demographic and occupational data, covering age, gender, country of birth, employment nature, socioeconomic status of the clinics' locale, and patients' population served. Information regarding any relevant post-medical school ethical-legal training received was also gathered.

*Statistical Analyses***:** The collected data were analyzed using t-tests or ANOVA for comparison of responses across groups, with a significance threshold set at *p* < 0.05. Fisher’s exact test was employed to examine the association between categorical variables.

## Results

177 Israeli Obstetricians and Gynecologists responded to the survey (henceforth referred to as the full sample; Due to the snowball method, it is impossible to assess the response rate). Of the full sample, 132 (74.58%, referred to as the "exposure group") affirmed that "a minor (up to the age of 18) has come to their clinic in the past year, for the purpose of receiving contraceptives".

The participants had an average age of 51 years, with 65% identifying as female and 35% as male. A majority (74%) were born in Israel, and 80% graduated from medical schools in Israel. Regarding experience, 60% of participants were senior gynecologists with over 10 years of experience, 11% were seniors with 5–10 years of experience, 21% were young seniors with less than 5 years of experience, and 8% were residents. Approximately one-third of the participants were self-employed, another third held salaried positions, and the remaining third had a combination of both self-employment and salaried positions. The majority primarily treated Jewish women and worked with patients of medium–high and medium–low socioeconomic statuses (47% and 39%, respectively).

In terms of education regarding the provision of medical treatment to minors, 78% of participants reported never receiving such education, 18% attended a lecture on the topic at a conference, and less than 4% read about it.

As presented in Supplement 1, there are some significant demographic differences between the exposure and non-exposure sample groups. Members of the exposure group are older, more senior, and tend to be either self-employed or hold a salaried job alongside self-employment. The exposure group is also characterized by higher prevalence of high-socioeconomic work settings serving mostly Jewish cliental.

Gynecologists in the exposure sample (n = 132) treated between 1 and 5 contraceptives-seeking minors over the past year (18% of exposed participants), to between 20 and 50 (24% of exposed participants) and 16% of exposed participants treated more than 50 contraceptives-seeking minors during the past year.

When asked about situations in which contraceptives-seeking minors refused parental involvement, the majority of exposed gynecologists (63%) reported that this occurred “sometimes,” while 27% indicated that they “never” faced such instances. A smaller proportion (9.2%) stated that this happened “almost always.”

Gynecologists were asked if, in cases where minors refused parental involvement, they agreed to prescribe. A significant majority (74%) reported doing so “almost always,” while 20% indicated that they “never” prescribed in such circumstances, and 5.9% responded that they did so “sometimes.” As for documentation practices, 93% of exposed gynecologists “almost always” documented the prescription of contraceptive pills to minors in their medical files. However, when it came to documenting *the reasons* for prescribing without parental involvement, only 37% of respondents reported doing so “almost always,” with 40% indicating that they “never” documented their considerations, and 23% doing so “sometimes.” In terms of explaining to the minor that the prescription and discussion are documented in her medical file, 75% of gynecologists reported doing so “almost always,” 15% “never,” and 10% “sometimes.” (See Supplement 2 for a detailed breakdown of responses to various items related to clinical practice).

Among the exposed group (n = 132), 37% believed that there is no legal requirement to involve parents in the process. Others held the view that the obligation for parental consent varies with the age of the minor: 22% believed it is applicable only if the minor is under 14 years old, 15% if she is under 16, and less than 8% assumed a legal obligation for parental involvement in all cases of minors under the age of 18. Nineteen percent acknowledged uncertainty about whether there is a legal obligation to involve parents.

We sought to investigate whether gynecologists treating minors align their practices with their interpretation of the law. Table 2 displays the cross-tabulation of participants' responses regarding their willingness to prescribe contraceptives alongside their views on legal requirements pertaining to parental consent. Notably, we observed a consistent percentage of approximately 20% (ranging from 19 to 22%) of gynecologists who consistently refrain from prescribing without parental consent, irrespective of their interpretation of legal obligations. In parallel, approximately 70% (67–77%) of those who almost always prescribe, do so regardless of their perception of parental consent requirements.

**Table 2:** Perception of parental consent requirements and prescription practices

	Prescribes to minors			Total	*p* value^*1*^
	Almost always	Sometimes	Never		0.9
Age dependent	33 (77%)	2 (4.7%)	8 (19%)	43 (100%)	
Always required	6 (67%)	1 (11%)	2 (22%)	9 (100%)	
Does not know/never required	47 (72%)	4 (6.2%)	14 (22%)	65 (100%)	
Total	86 (74%)	7 (6.0%)	24 (21%)	117 (100%)	

^1^Fisher’s exact test

We also explored the connection between gynecologists' views on parental access to their minor child's medical information and their readiness to prescribe contraceptives to minors without parental awareness. No statistically significant correlation was identified between those who prescribed to minors and those who did not, based on their perceptions of parents' access to their child's data. (both perceptions regarding legal requirements and their correlation with practices are further detailed in Supplement 3).

Participants were asked to rate their agreement with several professional-ethical stances listed in Table 3.

**Table 3:** Support of stances by tendency to prescribe

Stance item	GTM's who always prescribe w/o parental inv	GTM's who occasionally prescribe w/o parental inv	GTM's who never prescribe w/o parental inv	*p* value
There are cases when it is right to give pills to a minor without the knowledge of her parents	4.85 (0.39)	4.29 (1.50)	4.38 (1.06)	0.003
It is better for the minor to receive pills without the knowledge of her parents, than for her to become pregnant	4.86 (0.44)	4.43 (1.13)	4.46 (1.02)	0.013
Giving pills to a sexually active minor is in her best interest, even if it is without her parents' knowledge	4.78 (0.49)	4.71 (0.49)	4.29 (1.08)	0.007
Minors should be allowed to choose whether to allow their parents to see the part of their medical file that deals with sexual and sexual issues	4.35 (0.91)	4.29 (1.11)	3.79 (1.18)	0.05
The fact that the minor's record is exposed to the parents prevents me from giving the minor good treatment of sexual and sexual issues	3.01 (1.48)	3.43 (1.40)	2.74 (1.51)	0.5
It can't be that parents didn't know that their underage daughter was taking pills	1.93 (0.99)	2.43 (0.79)	2.38 (1.17)	0.1
Giving pills to a minor encourages her to have sex	1.70 (0.83)	1.57 (0.53)	1.79 (1.14)	0.8
Teenage/extramarital sex is inappropriate	1.68 (0.95)	2.00 (1.15)	1.50 (0.66)	0.4
I am not willing to risk legal action, so I will not give pills to a minor without the knowledge and consent of a parent	1.60 (0.72)	2.14 (1.07)	2.22 (1.38)	0.01
In cases where it is in the best interest of the minor patient, I will give her a prescription for pills even without her parents knowing about it	4.45 (1.01)	3.71 (1.60)	3.58 (1.38)	0.003

The following three statement received the highest agreement scores, regardless of prescription practices: "There are cases when it is right to give pills to a minor without the knowledge of her parents"; "It is better for the minor to receive pills without the knowledge of her parents, than for her to become pregnant"; "Giving pills to a sexually active minor is in her best interest, even if it is without her parents' knowledge".

Yet, we found that gynecologist who were willing to prescribe without parental involvement demonstrated significantly higher support for these three statements (*P* = 0.003, 0.013 and 0.007 respectively), as well as for the following two statements: " Minors should be allowed to choose whether to allow their parents to see the part of their medical file that deals with sexual and sexual issues" (*P* = 0.05) and “In cases where it is in the best interest of the minor patient, I will give her a prescription for pills even without her parents knowing about it” (*p* = 0.003).

Gynecologists who refuse to prescribe without parental permission demonstrated significantly higher support for the following statement: "I am not willing to risk legal action, therefore I will not give pills to a minor without parental consent" (*p* = 0.01).

Participants were also tasked with evaluating the significance of factors influencing their decision to either prescribe or withhold prescription of contraceptives to minors without parental consent. As seen in Table 4, the consideration acknowledged as the most influential by all participants, regardless of their prescription practices, is "The risk to the health of the minor as a result of having sex without pills". Yet, again, gynecologists who never opt for prescription, though supportive of this consideration, were significantly less affected by it (*p* = 0.035).

**Table 4:** Factors influencing decision to prescribe

Consideration	Almost always, N = 87^1^	Sometimes, N = 7^1^	Never, N = 24^1^	*p* value^2^
Parents right to know	2.29 (1.04)	3.00 (1.29)	2.61 (1.37)	0.2
Fear of damaging my relationship with the minor's parents	2.15 (1.05)	2.57 (1.27)	2.26 (1.21)	0.6
Fear of a violent/harsh reaction by parents towards me	2.61 (1.44)	2.14 (0.90)	2.35 (1.37)	0.5
Fear of legal action	2.06 (1.07)	2.17 (0.98)	2.57 (1.50)	0.2
Age of the minor	3.56 (1.31)	4.00 (1.10)	3.65 (1.11)	0.7
The minor's ability to understand the significance of the decision, the implications and risks	4.38 (1.05)	4.00 (1.15)	3.95 (1.00)	0.2
Does the minor have a rational reason for refusal (as opposed to just not wanting the parents to know)	3.05 (1.41)	3.71 (1.38)	3.45 (1.30)	0.3
Rights and dignity of the minor	4.48 (0.78)	4.29 (0.95)	4.14 (0.89)	0.2
My ability to verify her medical background and contraindications to pills without parental involvement	4.24 (1.08)	4.00 (1.29)	4.05 (1.09)	0.7
The risk to the health of the minor that may be caused by taking the pills	4.16 (1.21)	3.57 (1.81)	3.87 (1.25)	0.3
The risk to the health of the minor as a result of having sex without pills	4.72 (0.81)	4.71 (0.76)	4.22 (0.85)	0.035
Fear of a violent/harsh reaction by the parents towards the minor if they knew that she is taking pills	3.65 (1.30)	3.14 (1.21)	3.09 (1.62)	0.2
The desire to preserve the minor in a therapeutic relationship with me	3.27 (1.31)	4.00 (1.26)	2.57 (1.44)	0.026
How supportive the parents really are, and how valuable it is for them to know that the minor is having sex and taking pills	3.41 (1.15)	3.14 (1.21)	3.43 (1.24)	0.8
Existence/absence of other adult support resource for the minor (aunt, older sister, etc.) instead of her parents	3.55 (1.24)	3.14 (1.21)	3.83 (0.98)	0.4

^1^Mean (SD)

^2^One-way ANOVA

Next in line, as influential considerations, were "The Rights and dignity of the minor" and "the minor's ability to understand the significance of the decision, the implications and risks", with no statistically significant difference between the groups. A statistically significant difference was found regarding the desire to "preserve the therapeutic bond with the minor", which was less relevant to the decisions taken by those who never prescribe without parental consent (*p* = 0.026).

Fear of damaging one's relationship with the minor's parents, Fear of a violent/harsh reaction by parents and Fear of legal action were viewed as the least relevant consideration. Concerns about facing legal consequences are a somewhat more pertinent factor for participants who never prescribe, though not to a statistically significant extent.

**Fig. 1:**
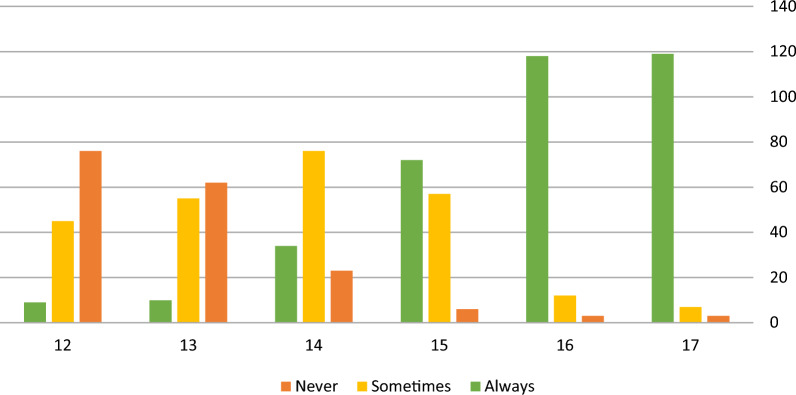
Appropriate age-threshold for confidential prescription. * X = age, Y = number of respondents

Lastly, participants were asked about the age at which they deemed it (ethically) appropriate, if at all, to prescribe contraceptive pills to a minor without involving or informing her parents. As described in Fig. 1, Most participants identified the age of 15 as the threshold for consistently prescribing contraceptives to minors without parental involvement or consent, while many believe it can be acceptable in certain cases between the ages of 14 and 15.

### Limitations

Our study employed a non-probability snowball sampling approach of Israeli OBGYNs, who were contacted through professional mailing lists, email, WhatsApp and social media groups.

The survey was answered by 177 OBGYNs, comprising 14% of the 1245 members The Israeli Association of Obstetrics and Gynecology (Based on the associations website), and about 9% of all OBGYNs registered in Israel (as of March 2024, based on the MoH's registration database).

Yet, as evident by the prevalence of self-employment in our sample, our sample mainly consist of OBGYNs working in community clinics, minded to prescribing contraceptives, which is the subsection of OBGYNs of concern here.

There are more Israeli born and educated women in our sample than there are in the general OBGYNs population in Israel, and many of them are highly experienced veteran physicians (35% with over 20 years of expertise). This may attest to both selection and response bias.

Furthermore, we recognize that family physicians and pediatricians in Israel also receive requests for prescribing contraceptives to minors. This has not been explored here and warrants further research.

## Discussion

Amongst the sampled gynecologists, there is a spectrum of experiences and practices concerning the prescription of contraceptives to minors. Senior, self-employed (either exclusively or alongside salaried positions) gynecologists, as well as those working in high socioeconomic—mostly Jewish serving settings, were more likely to encounter contraceptive seeking minors, with differing numbers of under-age patients. Members of this exposure group approached situations where minors declined parental involvement in various ways. Additionally, documentation practices varied among practitioners.

The study aligns with the literature, indicating that many minors seek prescription of contraceptives, occasionally expressing a preference for parental non-involvement. It is important to note that minors may receive contraceptive pills as medication for indications other than birth control, such as alleviating heavy menstrual bleeding or treating moderate acne. However, on these occasions this is given not at the request of the minor, nor her parents, but as a clinical recommendation by the physician. Thus, those medical interactions are not within the scope of this research, and the discussion regarding minors' confidentiality is irrelevant there.

Our research suggests a varied response to such requests, with no discernible connection between the inclination to prescribe without parental involvement and demographic factors, nor perception of parental accessibility to online medical data. Likewise, no connection was found between perceptions of legal obligations regarding parental involvement and actual practices: many of those of who almost always prescribe, do so regardless of their perception of parental consent requirements, and vis-versa.

On the other hand, a correlation was identified between medical practitioners' practices and their attitudes toward the importance of the therapeutic relationship, the rights of minors, and concerns about legal repercussions. In the decision-making process of prescribing pills to minors without parental involvement and consent, most participants place significant emphasis on the minor's rights, her ability to understand the implications of taking pills, the availability of reliable and comprehensive medical information, the potential risks associated with pill consumption, and the potential risks to the minor if she engages in sexual activity without using pills. Yet, the preservation of the therapeutic relationship with the minor holds greater importance for those who prescribe pills compared to those who do not, while the fear of potential legal action is a more significant consideration for those who opt not to prescribe pills, as opposed to those who agree to do so.

Section 6 of the Legal Capacity Law permits a minor to consent to treatment independently, without the lack of parental informed consent invalidating the action. This provision applies in contexts and circumstances where such actions are typically undertaken by minors of similar age on their own. As also attested to in the Ministry of Health's position from 1997 [15], the determination of whether, and at what age, minors commonly request contraceptives and are mature enough to do so, is best answered by the physicians who handle these requests.

While existing literature suggests that most requests for contraception consultations occur at the age of 15 and above, our findings reveal that most doctors tend to prescribe to 15-year-old girls and, in some cases, even to those as young as 14, with considerations of their maturity taken into account. It is important to note, that age in-of-itself has been found to be of less importance in the decision to prescribe confidentially, as compared to considerations of the minor's maturity and her rights and dignity, alongside the need to protect her health. This observation supports the argument that a girl aged 15 or older seeking contraceptive pills from a gynecologist falls within the scope of Article 6 of the Israeli legal competency and guardianship Law allowing physician to prescribe based on the mature child's consent alone.

The age of 15, as a watershed line for what "minors tend to do on their own", is further supported by the WHO-Israel HBSC report 2019. This report indicates that 85% of surveyed Jewish girls reported experiencing their first full sexual encounter at or after the age of 15 (30% at 15, and 55% at 16 or later). Only 9% reported engaging in full sexual activity at 14, and 1.5% at ages 12 and 13.

This age threshold also aligns with previous regulatory efforts in Israel aimed at defining the legal status and rights of minors in healthcare contexts. Notably, the 2014 Ministry of Health memorandum, issued to amend the Patient's Rights Law [23], underscores this alignment. The memorandum's objective is to grant autonomy to minors based on their developmental stage, considering both age and maturity, while concurrently safeguarding parental responsibility in line with the welfare and rights of the minor.

Regarding medical interventions related to sexuality, the memorandum delineates a "mature minor" as one who has reached the age of 14, unless the attending healthcare provider assesses that the minor has not yet attained maturity or lacks the capacity to comprehend the implications of making medical decisions without the consent of a legal guardian. Under the memorandum's provisions, contraceptive pills may be administered to a minor upon request, even without obtaining informed consent from their legal representative. This is contingent upon the healthcare provider engaging in a discussion with the minor regarding the importance of involving their representative in the decision-making process, furnishing the minor with pertinent medical information, ensuring the minor comprehends the information provided, and obtaining the minor's consent for treatment. Furthermore, as per Section 13d, information concerning sexuality, encompassing sexually transmitted diseases, sexual injuries, contraception, childbirth, termination of pregnancy, sexual relations, sexual identity, and gender, must not be divulged to the minor's parents without their explicit consent.

Although the memorandum's text was subsequently integrated into various private bills, such as the Patient Rights Bill P/966/24 of 2021, it has yet to progress into legislation [24].

### Policy recommendations

The findings from this study underscore the urgent need for comprehensive legal reform and the development of clear professional guidelines regarding the prescription of contraceptives to minors in Israel. The current legal ambiguity, combined with the absence of formal guidelines, contributes to inconsistent practices and uncertainty among healthcare providers, placing both minors and healthcare providers in a challenging position.

A critical step forward would be legislative reform to provide clear legal parameters for the prescription of contraceptives to minors. The new legislative framework should explicitly permit the prescription of contraceptives to minors aged 14 and above without requiring parental consent, contingent upon the healthcare provider’s assessment of the minor’s maturity and understanding of the implications. Such legislation would better serve minors health needs as well as align Israeli law with international standards that recognize the evolving capacities of adolescents, granting them greater autonomy over their reproductive health.

In addition, it is imperative to develop professional guidelines that clarify the ethical and legal considerations pertinent to the decision whether to prescribe contraceptives to minors. These guidelines should provide concrete instructions on how to handle cases where minors refuse parental involvement, including the need for evaluation of a minor’s capacity to consent, and proper documentation practices, ensuring that healthcare providers are equipped to make informed decisions that prioritize the minor’s health and rights.

Moreover, a national effort should be made to improve the education and training of obstetricians and gynecologists on the legal and ethical aspects of treating minors. This training should not be limited to sporadic lectures or individual reading materials but should be incorporated into the standard residency curriculum, including mandatory participation in clinical rounds in community gynecology clinics. Online modules, case studies, and decision-support tools embedded in electronic medical records could provide accessible and practical resources for healthcare providers. By ensuring that all professionals are well-informed, we can promote consistent and ethically and legally sound practices that safeguard the health and rights of minors.

## Conclusions

This study highlights the significant gaps in both the legal framework and the training of Israeli obstetricians and gynecologists regarding the prescription of contraceptives to minors. The majority of gynecologists in our study support prescribing contraceptives to minors, particularly those aged 15 and older, without parental involvement, yet they operate within a legal gray area that lacks clear guidelines. We urge Israeli regulators to formally acknowledge the needs, capacities, and rights of minors by enacting legislation that provides clear legal standing for minors to access contraceptives confidentially.

In the absence of comprehensive regulations, our findings support the argument that confidential prescriptions for minors aged 15 and above should be recognized as a legal action commonly undertaken by minors of that age, as outlined in Article 6 of the Israeli Legal Competence and Guardianship Law. This legal recognition should be accompanied by professional guidelines that clarify the responsibilities of healthcare providers in such cases.

Finally, the lack of formal training in managing cases where minors refuse parental involvement underscores the need for targeted educational initiatives. These initiatives should equip healthcare providers with the tools they need to navigate the complex legal and ethical issues surrounding the reproductive health of minors. By addressing these gaps, Israel can move toward a more coherent and ethically sound framework for adolescent reproductive care.

## Supplementary Information

Supplementary Material 1.

Supplementary Material 2.

Supplementary Material 3.

## Data Availability

The datasets generated during the current study available from the corresponding author on reasonable request.
