# Supplementary material for: Improving contraceptive care for minors in Israel: practice, policy, and training gaps among OBGYNs
Source: Isr J Health Policy Res. 2024 Sep 26;13:52. doi: 10.1186/s13584-024-00638-4 (PMC11425984; doi:10.1186/s13584-024-00638-4)
Supplement: Supplementary file 1 — Supplementary Material 1. [file 13584_2024_638_MOESM1_ESM.docx]

**Supplement 1.** Participant's Demographics

| **Characteristic** | | **Exposure**,  N = 132*^1^* | **Non exposure**,  N = 45*^1^* | **P-value** |
| --- | --- | --- | --- | --- |
| age |  | 52.2 (11.3) | 45.9 (10.5) | 0.0013 |
|  | Unknown | 4 | 2 |  |
| sex | Female  male  Unknown | 85 (64.9%)  46 (35.1%)  1 (0.8%) | 29 (64.4%)  16 (35.6%)  0 | 0.9574 |
| Place of birth | Israel  Former USSR  Eastern Europe  USA / Canada  Western Europe  South America  other  Unknown | 93 (71.0%)  17 (13.0%)  8 (6.1%)  6 (4.6%)  5 (3.8%)  1 (0.8%)  1 (0.8%)  1 | 38 (84.4%)  4 (8.9%)  0  2 (4.4%)  0  1 (2.2%)  0  0 | 0.3474 |
| Age of youngest child | 0-11 years  12-14 years  15-17 years  18 and up  No children  Unknown | 50 (38.2%)  9 (6.9%)  14 (10.7%)  53 (40.5%)  5 (3.8%)  1 | 25 (56.8%)  5 (11.4%)  2 (4.6%)  10 (22.7%)  2 (4.6%)  1 | 0.100 |
| Employment | Salaried  salaried and self employed  Self employed  Unknown | 30 (23.1%)  48 (36.9%)  52 (40.0%)  2 | 22 (52.4%)  10 (23.8%)  10 (23.8%)  3 | 0.0015 |
| Patients' population | Mostly Arab  Mixed  Mostly Jewish  Unknown | 0 (0%)  43 (33.1%)  87 (66.9%)  2 | 3 (7.0%)  17 (39.5%)  23 (53.5%)  2 | 0.0056 |
| Patients' socioeconomic status | Low socioeconomic  Medium Low  Medium High  High socioeconomic  Unknown | 3 (2.3%)  42 (32.6%)  68 (52.7%)  16 (12.4%)  3 | 2 (4.6%)  25 (56.8%)  13 (29.5%)  4 (9.1%)  1 | 0.0221 |
| Place of graduation from medical school | Israel  Eastern Europe  Western Europe, USA, Canada  Middle East  other  Unknown | 106 (81.5%)  13 (10.0%)  7 (5.4%)  1 (0.8%)  3 (2.3%)  2 | 36 (80.0%)  3 (6.7%)  2 (4.4%)  1 (2.2%)  3 (6.7%)  0 | 0.5673 |
| Seniority (years) | Resident  ≤ 5  5-10  10-20  20+  Unknown | 2 (1.6%)  32 (24.8%)  11 (8.5%)  34 (26.4%)  50 (38.8%)  3 | 12 (26.7%)  5 (11.1%)  8 (17.8%)  9 (20.0%)  11 (24.4%)  0 | < 0.001 |
| Did you participate in any training regarding treating of minors without parental involvement? | no training  Yes, conference  Yes, written procedure  Unknown | 95 (73.0%)  30 (23.1%)  5 (3.8%)  2 | 42 (93.3%)  2 (4.4%)  1 (2.2%)  0 | 0.0153 |
|  | *^1^* Mean (SD); n (%) | | |  |
