# Supplementary material for: Improving contraceptive care for minors in Israel: practice, policy, and training gaps among OBGYNs
Source: Isr J Health Policy Res. 2024 Sep 26;13:52. doi: 10.1186/s13584-024-00638-4 (PMC11425984; doi:10.1186/s13584-024-00638-4)
Supplement: Supplementary file 2 — Supplementary Material 2. [file 13584_2024_638_MOESM2_ESM.docx]

**Supplement 2** **– Clinical reality among the exposure group**

| **Characteristic** | **N = 132**^1^ |
| --- | --- |
| How many contraception consultations for minors did you perform during the past year? |  |
| 1-5 | 24 (18%) |
| 5-10 | 25 (19%) |
| 10-20 | 30 (23%) |
| 20-50 | 31 (24%) |
| 50+ | 21 (16%) |
| Unknown | 1 |
| In how many cases did the minor refuse parental involvement? |  |
| always/almost always | 12 (9.2%) |
| sometimes | 83 (63%) |
| never | 36 (27%) |
| Unknown | 1 |
| When a minor refused parental involvement, did you actually prescribe contraception without parental consent? |  |
| always/almost always | 87 (74%) |
| sometimes | 7 (5.9%) |
| never | 24 (20%) |
| Unknown | 14 |
| In cases in which you prescribed contraceptives without parental consent, did you document the prescription in the minor’s health file |  |
| always/almost always | 104 (93%) |
| sometimes | 2 (1.8%) |
| never | 6 (5.4%) |
| Unknown | 20 |
| In cases in which you prescribed contraceptives, did you document the reasons for prescribing without parental consent in the minor’s health file? |  |
| always/almost always | 41 (37%) |
| sometimes | 26 (23%) |
| never | 44 (40%) |
| Unknown | 21 |
| In cases in which you prescribed contraceptives without parental involvement, did you explain to the minor that the prescription is documented in her health file? |  |
| always/almost always | 83 (75%) |
| sometimes | 11 (10%) |
| never | 16 (15%) |
| Unknown | 22 |
| ^1^n (%) | |
