# Supplementary material for: Improving contraceptive care for minors in Israel: practice, policy, and training gaps among OBGYNs
Source: Isr J Health Policy Res. 2024 Sep 26;13:52. doi: 10.1186/s13584-024-00638-4 (PMC11425984; doi:10.1186/s13584-024-00638-4)
Supplement: Supplementary file 3 — Supplementary Material 3. [file 13584_2024_638_MOESM3_ESM.docx]

**Supplement 3 – Connection between Perceptions of legal requirements and prescription practices**

***3.1 – Perception of legal requirements amongst the exposure group***

| **Characteristic** | | **Treats minors**, N = 132*^1^* |
| --- | --- | --- |
| Legal requirement to involve parents | No legal obligation      Only if under the age of 14      Only if under the age of 16      Yes (at any age of a minor)      Don't know      no response | 48 (37%)  29 (22%)  19 (15%)  10 (7.6%)  25 (19%)  1 |
| Parents can view visit summary | yes      no      Don't know      no response | 48 (37%)  47 (36%)  36 (27%)  1 |
| Parents can view contraceptive prescriptions | yes      no     Don't know      no response | 58 (44%)  32 (24%)  41 (31%)  1 |
| Parents can view purchase history | yes      no      Don't know      no response | 71 (55%)  19 (15%)  39 (30%)  3 |

***Table 3.2 – Perception of parental data access and prescription Practices***

**Table** **3.2.1: access to visit** **summary**

|  | Parents can view visit summary | |  | |
| --- | --- | --- | --- | --- |
|  | yes | no/doesn't know | Total | p-value^1^ |
| Prescribes to minors |  |  |  | 0.8 |
| yes | 34 | 58 | 92 |  |
| no | 9 | 14 | 23 |  |
| Total | 43 | 72 | 115 |  |
| ^1^Pearson's Chi-squared test | | | | |

**Table 3.2.2** **: access to contraceptive prescription**

|  | Parents can view contraceptive prescriptions | |  | |
| --- | --- | --- | --- | --- |
|  | yes | no/doesn't know | Total | p-value^1^ |
| Prescribes to minors |  |  |  | 0.5 |
| yes | 43 | 49 | 92 |  |
| no | 9 | 14 | 23 |  |
| Total | 52 | 63 | 115 |  |
| ^1^Pearson's Chi-squared test | | | | |

**Table** **3.2.3: access to contraceptives purchase history**

|  | Parents can view purchase history | |  | |
| --- | --- | --- | --- | --- |
|  | yes | no/doesn't know | Total | p-value^1^ |
| Prescribes to minors |  |  |  | 0.13 |
| yes | 48 | 44 | 92 |  |
| no | 16 | 7 | 23 |  |
| Total | 64 | 51 | 115 |  |
| ^1^Pearson's Chi-squared test | | | | |
